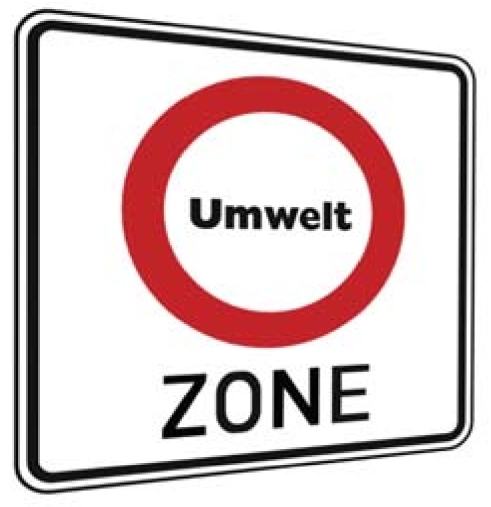# The Beat

**Published:** 2008-02

**Authors:** Erin E. Dooley

## Antibacterial Alters Hormone Activity

In a study published online 29 November 2007 ahead of print in *Endocrinology*, researchers at the University of California, Davis, report that the commonly used antibacterial chemical triclocarban (TCC) acts as an endocrine disruptor by a previously unreported mechanism. In human cells, the researchers found that TCC increased gene expression that is normally regulated by testosterone. In male rats, they found that testosterone-dependent organs grew abnormally large after the rats were fed TCC. Until this study, endocrine disruptors had only been found to block the effects of hormones. TCC has been used in a variety of household and personal care products for more than 45 years.

**Figure:**
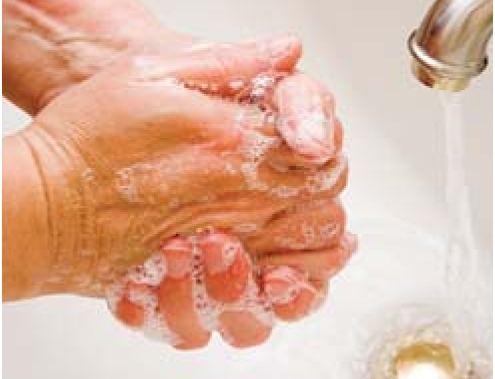


## Raising Awareness of Nurse Health

On 11 December 2007, the Environmental Working Group released the results of a survey of occupational health among nurses. More than 1,500 U.S. nurses participated in the online survey, which assessed workplace exposures to common hazardous substances and other agents as well as the health of nurses’ children. As had been seen in earlier studies, nurses who experienced routine high exposures to sterilizing and cleaning chemicals, residues from drug preparation, anesthetic gases, radiation, and other hazardous agents reported higher rates of asthma, miscarriage, and certain cancers, and their children had higher rates of cancer and birth defects (especially musculoskeletal defects). The goal of the survey was to encourage hospitals to minimize risks to nurses and inspire further study.

## Minnesota Bans Mercury in Beauty Products

On 1 January 2008, Minnesota became the first state to ban mercury from cosmetics such as mascara, eyeliner, and skin-lightening creams. Current federal regulations allow up to 65 ppm mercury to be used in cosmetics as a preservative and germicide. The cosmetics industry claims the levels of mercury found in cosmetics pose little risk to human health, but an August 2005 WHO policy paper asserts that “studies suggest that mercury may have no threshold below which some adverse health effects do not occur.” Skin-lightening creams could pose more of a health risk as people generally apply relatively large amounts of them over sizeable areas of their bodies.

**Figure:**
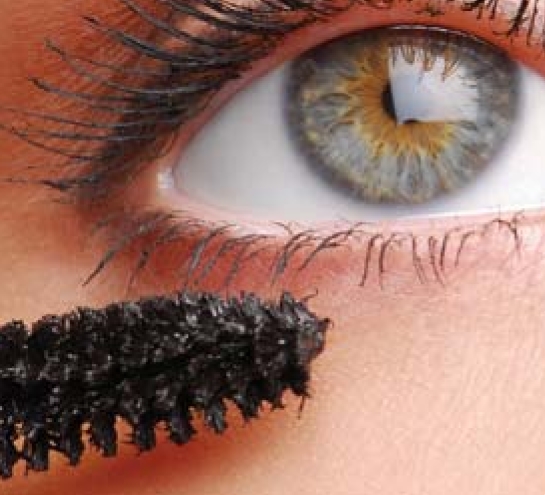


## Hops for Health?

Xanthohumol, a flavonoid compound found in the hop plant, is a powerful antioxidant that can reduce the activity of cancer-causing cytochrome P450 enzymes. Earlier studies have found that xanthohumol can kill cultured breast, colon, ovarian, and prostate cancer cells, and can also reduce the oxidation of LDL (“bad”) cholesterol. Now an article in the November 2007 issue of *Apoptosis* shows that xanthohumol induces apoptosis in adipocytes and inhibits adipogenesis in maturing preadipocytes, leading its authors to conclude the compound could be useful as an antiobesity agent. Also in autumn 2007, the German company TA-XAN AG launched XAN Wellness, the first product to employ a patented process that supposedly boosts the health-promoting properties of xanthohumol.

**Figure:**
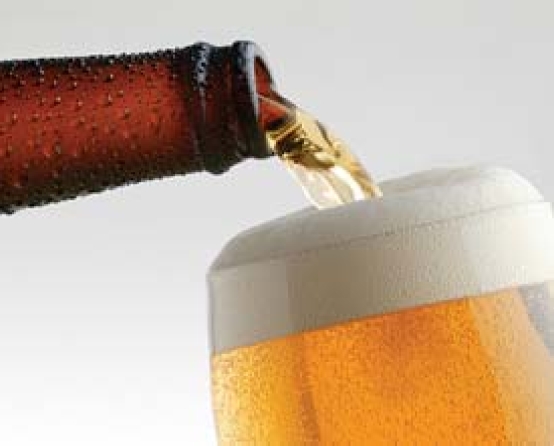


## New Tactic for Taming Invasive Species

Each year, in response to rules adopted in 2004 by the International Maritime Organisation (IMO), the shipping industry spends billions of dollars to remove exotic species from the 3–5 billion tons of water transported in ballast tanks. A South African company, Resource Ballast Technologies, is on track to commercialize a new system for this purpose that, unlike some conventional methods, does not use harmful chemicals. Instead, it combines ozone and ultrasonic radiation to kill organisms. The small unit fits on existing ballast discharge pipes. The company has submitted its technology to the IMO for approval.

## Germany Takes Aim at Auto Emissions

On 1 January 2008, the German cities of Berlin, Cologne, and Hanover witnessed the rollout of a new program to reduce air pollution in city centers. In those cities, and in 17 more to be added during the year, cars are required to display color-coded stickers that reflect how much fine particulate pollution they emit, as determined by an authorized testing facility. Cars that emit too much particulate matter will not be allowed in central “environmental zones,” or *umweltzones*, of participating cities. Drivers who enter city centers without a sticker will receive a fine of up to US$60 dollars and a point on their driver’s license. The new laws are part of an effort to help Germany meet new European Union standards for air quality.

**Figure:**